# Roles of Interfacial Modifiers in Inorganic Titania/Organic Poly(3-hexylthiophene) Heterojunction Hybrid Solar Cells

**DOI:** 10.3390/nano12050820

**Published:** 2022-02-28

**Authors:** Arumugam Pirashanthan, Thirunavukarasu Kajana, Dhayalan Velauthapillai, Yohi Shivatharsiny, Said Bentouba, Punniamoorthy Ravirajan

**Affiliations:** 1 Clean Energy Research Laboratory, Department of Physics, University of Jaffna, Jaffna 40000, Sri Lanka; tskajana@gmail.com (T.K.); pravirajan@univ.jfn.ac.lk (P.R.); 2 Faculty of Engineering, Western Norway University of Applied Sciences, 5020 Bergen, Norway; said.bentouba@hvl.no; 3 Department of Chemistry, University of Jaffna, Jaffna 40000, Sri Lanka; yshiva@univ.jfn.ac.lk

**Keywords:** hybrid solar cells, interfacial modifiers, Titanium dioxide, Poly(3-hexylthiophene), working principle, self-assembled monolayers, insulating/semiconducting layers, carbonaceous materials, small molecule sensitizers, charge transport, light harvesting properties

## Abstract

Hybrid Titanium dioxide/Poly(3-hexylthiophene) heterojunction solar cells have gained research interest as they have the potential to become cost-effective solar technology in the future. Limited power conversion efficiencies of about 5–6% have been reported so far, and an enhancement in efficiency was achieved through the engineering of the interface between Titanium dioxide (TiO_2_) and Poly(3-hexylthiophene) (P3HT). Evolution of this solar cell technology is relatively slow-moving due to the complex features of the metal oxide-polymer system and the limited understanding of the technology. In this review, we focus on recent developments in interface modified hybrid Titanium dioxide/Poly(3-hexylthiophene) solar cells, provide a short discussion on the working principle, device structure with interface modifiers, and summarize various types of interface modifiers studied to enhance the photovoltaic performance of hybrid TiO_2_/P3HT heterojunction solar cells. Further, we discuss the key factors influencing the power conversion efficiency and the role of a variety of interface modifiers in this regard. Finally, the challenges and perspectives related to hybrid TiO_2_/P3HT heterojunction solar cells are also explored.

## 1. Introduction

Molecular electronic materials, such as dyes, conjugated polymers, and small molecules, are gaining much interest for applications in photovoltaics [1,2]. In this regard, the organic photovoltaics (OPVs) have attracted much attention due to their features such as low cost, flexibility, ease of fabrication, and large field of application areas [3,4,5,6,7,8,9]. In particular, hybrid and dye synthesized solar cells are the main focus in the field of OPVs [10,11,12,13]. Hybrid polymer/nanocrystalline solar cells are good and effective model systems to analyze nanostructured solar cell technologies. With fewer junctions compared to other OPVs, a small alteration in the material structures will result in vast variations in the performance parameters. Therefore, these hybrid solar cells are one of best model structures to study the performance of new dyes, perovskite materials, surface modifiers, and dopants. Herein, we have focused on recent enhancements in interface modifications carried out to enhance the efficiency of hybrid Titanium dioxide/Poly(3-hexylthiophene) solar cells.

The blend of conjugated polymers with nanostructured metal oxides represent promising candidates for hybrid solar cells since these exhibit high solar energy conversion with low cost [14,15,16]. Typically, hybrid solar cells are made up of a combination of both inorganic and organic materials [17,18,19]. They consist of a conjugated polymer as an electron donor and a nanocrystalline metal oxide as the electron acceptor [17,18,19]. Therefore, they have unique properties of inorganic semiconductors with the film-forming properties of the conjugated polymers [20,21,22,23,24,25]. Inorganic metal oxide semiconductors have their own relatively high electron mobility, high electron affinity, good thermal stability, facile exciton dissociation, and mechanical stability [26,27,28,29]. The nanoscale metal-oxide nanoparticle network provides a stable and transparent backbone for free carrier transport. Further, it can be synthesized as size-tunable nanoparticles with high absorption coefficients [30]. Likewise, organic materials are inexpensive and easily processable. Their properties can be tailored by chemical synthesis and molecular design. Conjugated polymers offer potential advantages, such as high hole mobility, low cost, facile synthesis via wet chemical processing, control of heterojunction morphology, and the potential for higher physical and chemical stabilities [30]. Moreover, the bandgap can be tuned by varying the size of the nanoparticles, which helps to tailor the absorption range [11]. Hence, suitable strategies are required to increase this attention by overcoming the limitations, such as the range of absorbance of organic materials, low efficiency, and poor stability [31].

In hybrid metal oxide polymer solar cells, the electron transfer from π-conjugated polymers (donor) into the nanoporous metal oxide (acceptor) produces a large proportion of charge carrier pairs across the donor/acceptor interface. Moreover, the conjugated polymers can be easily processed onto the surfaces of metal oxide nanoparticles, which facilitate efficient electron-hole pair creation through the enhanced interfacial area. However, the Coulombic attraction of bound charge carrier pairs limits the overall performance through prompted recombination at the interface [13,32]. The engineering of the metal-oxide polymer interface takes more attraction to improve the power conversion efficiency of hybrid solar cells [33].

To date, hybrid solar cells have been investigated with four major categories of inorganic materials, such as silicon, cadmium compounds, metal oxide nanoparticles, and low bandgap nanomaterials. Si, CdS, CdTe, CdSe, PbS, TiO_2_, ZnO, and ZnS are typical examples for inorganic semiconductors that exhibit unique electronic and optical features [34,35]. Several combinations in blending of organic and inorganic materials have been utilized, and the nanostructures of the semiconductor materials in the form of nanotubes or nanoparticles have shown to play a major role in enhancing the performance of hybrid solar cells by improving charge separation [30]. Further, selecting favorable acceptor materials with some important physical properties, such as solubility in a common solvent with the donor material, availability and cost of the material, capability to reach a balance between electron and hole mobilities, and the success of the nano-morphology of the donor/acceptor phases [34], also play a central role in the performance of the hybrid solar cells.

Photosensitizer/interface modifiers which are used in the donor-acceptor interface have been shown to improve the open-circuit voltage (*V_OC_*) and fill factor (FF) by suppressing surface recombination [36]. However, due to the high degradation rate of p-type organic material, it is difficult to find an ideal dye as a photosensitizer/interface modifier with suitable bandgap with less degradation [37]. The performance of polymer based solar cells mainly depends on the key factors of polymer infiltration, morphology, charge separation, charge recombination, and charge transport [14,17,38].The above factors can be improved with suitable strategies, such as modification and increasing the surface area of the electron collector structure or improving interfacial conductivity and light absorption through appropriate interface engineering methods, which improves the overall performance of the hybrid solar cell by increasing the light absorption [3,39,40,41].

The photoelectrode of the hybrid solar cells consists of a nanoporous wide bandgap semiconductor interconnected layer that is sensitized for the visible spectrum by a dye. The nanoporous semiconductor layer acts as a support layer for the dye molecules and a region for electron transport. There are several requirements for the selection of an efficient photoelectrode material. It should have sufficiently high surface area for dye adsorption, to facilitate the efficient excitation of electrons. The conduction band of the photoelectrode material should lie slightly below the excited state level of the sensitizer. The material should have high charge carrier mobility, should be easy to synthesis, and should be low cost and environmentally friendly. Moreover, they should have good electronic, photo-conducting, and luminescent properties. Titanium dioxide (TiO_2_) satisfies all the above requirements as an efficient photoelectrode compared to other semiconductors, such as ZnO, CdS, CdSe, CdTe, PbS, PbSe, Sb_2_S_3_ Cu_2_ZnSnSe_4_, Ag_2_S, AgInS_2_, etc., which were used in these hybrid solar cells [4,42,43,44,45]. TiO_2_ is a chemically stable, non-toxic, low cost material that provides controllable morphology and is available in large quantities [46,47,48,49,50,51]. It mainly exists in three different forms as rutile, anatase, and brookite [52,53].

In past decades, various polymer materials have been used in hybrid metal oxide polymer solar cells. Subsequently, they belong to two major types of polymers and their derivatives of Polythiophene (PT) and Poly(p-phenylene vinylene) (PPV). These polymers are promising for light-harvesting with a wider spectral band and it absorbs the solar radiation up to 650 nm. The polymer donor should have a small bandgap, enhanced packing structure, and high hole mobility in order to obtain an enhanced PCE of the device. The proximity of the LUMO energy of the polymer donor and the CB energy of metal oxide acceptor encourages electron transfer. Among other polymer donors reported in the literature, the homopolymer Poly(3-hexylthiophene) (P3HT) has been gaining much attraction due to its regular end-to-end packing arrangement of the side chain, which allows efficient π-π stacking of the conjugated backbones [54,55,56]. The high molecular weight with ultra-high purity and good chemical stability, higher hole mobility, and low optical band gap (1.8 eV) of P3HT are optimized for usages in the organic photovoltaic research and OPV devices [56,57,58,59,60]. P3HT has been extensively used as a semiconducting layer in organic thin-film, field-effect transistors (FETs), and solar cells.

Hybrid metal oxide/polymer solar cells provide the potential to study the science and to take the advantage of both TiO_2_ and P3HT as combined nanocomposites. This leads to combined spectral absorption of both TiO_2_ and P3HT in order to enhance the light-harvesting and carrier generation [23]. Subsequently, the dissociation of excited electrons takes place at the TiO_2_/P3HT interface. As discussed earlier, the charge recombination needs to be reduced with increased electron dissociation and charge separation. In order to facilitate proper electron dissociation and charge separation, interface engineering has been carried out between TiO_2_ and P3HT nanocomposite, and several proven strategies were reported in interface modified Hybrid TiO_2_/P3HT solar cells. Namely, a range of novel organic and inorganic materials, such as self-assembled monolayers, carbonaceous materials, inorganic insulating layers, and small molecule sensitizers, was reported in order to improve the TiO_2_/P3HT interface [13]. However, it has to be emphasized that there are still many ways to enhance the performances of hybrid TiO_2_/P3HT photovoltaic devices. A well-oriented hybrid TiO_2_/P3HT nanocomposite design with suitable interface modification will be one of the promising ways to produce enhanced hybrid TiO_2_/P3HT photovoltaic devices.

This review focuses on the recent progress witnessed in the field of hybrid Titanium dioxide (TiO_2_)/Poly(3-hexylthiophene) (P3HT) heterojunction solar cells mainly from the point of interface engineering. Therefore, for the benefit of completeness, we briefly describe the working mechanism of the proposed device structure with interface modifiers in Titania based hybrid polymer/nanocrystalline solar cells. By comparing various strategies and different types of interface modifiers with proposed nanostructures, we summarize the key factors influencing the photovoltaic power conversion efficiency and other relative photovoltaic parameters of hybrid TiO_2_/P3HT solar cells. Further, we briefly discuss different organic and inorganic interface modifiers and their role. Finally, we outline the challenges and perspectives for future improvements related to the realization of highly efficient hybrid solar cells.

**Figure 1:**
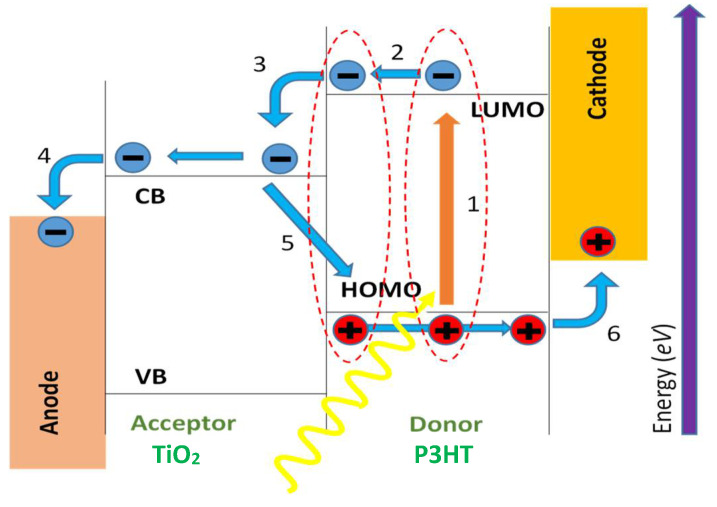
Principle process in hybrid metal oxide/polymer solar cell in an energy level diagram under short circuit conditions [39,61]. The step numbers represent as (1) light absorption ($\eta_{a}$) and Exciton creation ($\eta_{ex}$), (2) diffusion of the exciton to the interface between the metal oxide and polymer ($\eta_{diff}$), (3) electron-hole pair dissociation into free charge carriers ($\eta_{ed}$), (4) charge transport ($\eta_{tr}$), (5) recombination of charge and (6) charge collection ($\eta_{cc}$).

The graphical representation of the working principle of a metal oxide/polymer hybrid solar cell under short circuit conditions [39,61] is shown in Figure 1, and it has six major steps. The step numbers represent as (1) light absorption ($\eta_{a}$) and exciton creation ($\eta_{ex}$) (a photon with an energy $h\nu \geq E_{g}$ of polymer donor will absorbed), (2) diffusion of the exciton to the interface between the metal oxide and polymer ($\eta_{diff}$), (3) electron-hole pair dissociation into free charge carriers ($\eta_{ed}$), (4) charge transport ($\eta_{tr}$), (5) recombination of charge, and (6) charge collection ($\eta_{cc}$).

Upon the solar illumination, the photon energy will be absorbed by the active layer of the solar cells and it generates electron-hole pairs as excitons [62]. Thereafter, the holes and excited electrons need to be transported towards the anode and cathode electrodes respectively through their corresponding percolation pathways in order to have current flow through the fabricated solar cells. As illustrated, the donor material creates the exciton by absorbing the photon energy from the sunlight and allows the electron to be excited to the lowest unoccupied molecular orbit (LUMO) by leaving a hole from the highest occupied molecular orbit (HOMO). Next, the excited electron is dissociated from the LUMO level and transported to the conduction band (CB) of the acceptor material. The difference in the energy levels of CB and HOMO should be well matched to avoid recombination. This will lead to good charge collection through the anode and cathode [28]. Further, the percentage of the number of charge carriers collected at the electrode with the number of incident photons under short-circuit condition determines the external quantum efficiency (EQE) of the solar cells. The involvement of each of the six steps of the above mechanism highly influences the EQE of fabricated hybrid solar cells.

**Figure 2:**
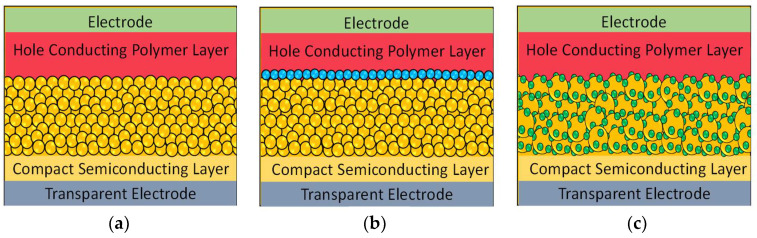
A proposed model of a device structure for nanoparticle based TiO_2_/P3HT hybrid solar cells and their interface modification. Depicted (**a**). TiO_2_ nanoparticle based mesoporous structure and P3HT nanocomposite without any interface modification, (**b**). TiO_2_ nanoparticle based mesoporous and P3HT nanocomposite structure with insulating layers, and (**c**). TiO_2_ nanoparticle based mesoporous and P3HT nanocomposite structure with small molecule interface modifiers.

Figure 2 illustrates the proposed model of the device structure for nanoparticle based TiO_2_/P3HT hybrid solar cells and their two major varieties of interface modifications. The transparent conducting electrode is crucial for a solar cell in order to transmit the incident light through itself to photon absorbing layers. The overall nanocomposite of the TiO_2_/P3HT hybrid solar cell is very thin, about one micrometer. Therefore, the P3HT can easily penetrate through TiO_2_ and overpenetration of P3HT may lead to contact with the transparent conductive electrode, and thus the junction of fabricated solar cells will function as a line but not as the solar cell. On this occasion, the compact semiconducting layer can act as a barrier in order to prevent the contact between P3HT and transparent conductive electrodes.

Figure 2a represents the TiO_2_ nanoparticle based mesoporous structure and P3HT nanocomposite without any interface modification. In past decades, there have been several inorganic and organic interface modifiers studied for TiO_2_/P3HT hybrid solar cells. However, all these interface modifiers can be specified in to two major groups, namely insulating layers and absorbing materials. Figure 2b,c represent the device structures for TiO_2_ nanoparticle based mesoporous and P3HT nanocomposite with insulating layers and small molecule based absorbing materials, respectively.

In this review, we have summarized the recent progress made and tactics used in interface modified hybrid TiO_2_/P3HT solar cells with a range of novel organic and inorganic materials, such as self-assembled monolayers, carbonaceous materials, inorganic insulating layers, and small molecule sensitizers. Further, we have summarized our review through Tables below based on the type of interface modifier and each table is divided into four columns in order to represent the device structure to indicate where the interface modifier is used, the role of interface modification or function of interface modifier on the photovoltaic performance, resultant energy conversion efficiency, and source of reference. The essence of this analysis will be of interest for researchers in order to understand the nanostructure of hybrid Tatiana based Poly(3-hexylthiophene) heterojunction solar cells. Furthermore, the analyzed parameters and findings will also be useful for direct implementations in hybrid perovskite solar cells and DSSCs.

## 2. Photovoltaic Performances

The cost-efficient hybrid polymer/nanocrystalline solar cells are promising models to study the effects of interfacial properties and film morphology on the performance of nanostructured solar cells [63]. They provide a valuable understanding of the charge-transfer processes at the donor–acceptor interface. Moreover, expertise gained with hybrid systems has proven to be valuable in improving the performance of all organic based solar cells as well as perovskite solar cells. In this Titanium dioxide (TiO_2_)/Poly(3-hexylthiophene) (P3HT) hybrid structure, the controllable morphological nature of nanoscale Titanium dioxide provides a stable and transparent network for free carrier transport. The thiophene rich P3HT is one of the promising polymer materials with higher hole-mobility to build hybrid nanostructured solar cells with TiO_2_. Although this system provides several advantages, the power conversion efficiency (PCE) of this hybrid structure is highly influenced by interfacial recombination. However, the interfacial recombination could be reduced by employing interface modifiers and hence enhance the overall PCE of the solar cells. In the literature, four major categories of interface modifiers, namely self-assembled monolayers (SAMs), inorganic insulating/semiconducting layers, carbonaceous materials, and dye molecules, have been studied in hybrid TiO_2_/P3HT solar cells.

### 2.1. Self-Assembled Monolayers (SAMs) as Interface Modifiers

Self-assembled monolayers (SAMs) are ordered arrays of organic molecules formed by the spontaneous absorption onto the TiO_2_ surface. The molecules or ligands that form SAMs are chemically functionalized in order to have a higher affinity to the surface. Various self-assembled monolayers (SAMs) reported in the literature are summarized in Table 1. Here, the different device structures studied and the effect of interface modifiers on the PV performance are discussed.

**Figure 3:**
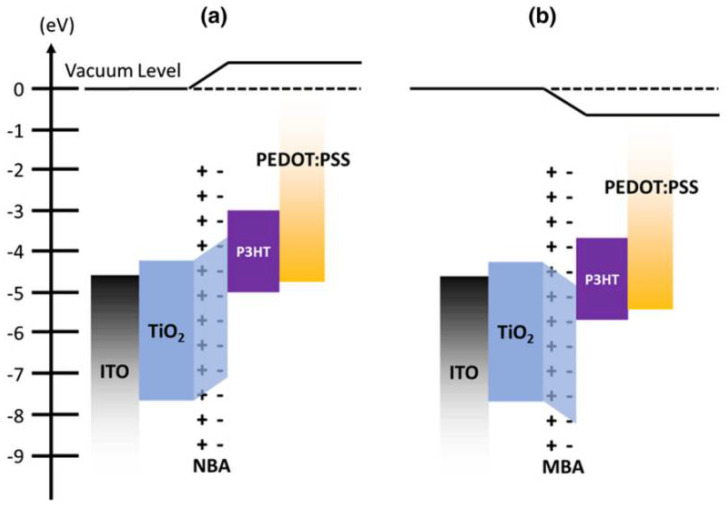
Energy band diagrams for energy level shifting of SAMs at the TiO_2_/P3HT interface of ITO/TiO_2_/SAMs/P3HT/PEDOT:PSS/Au device. NBA and MBA are 4-Nitro Benzoic Acid and 4-Methoxy Benzoic Acid, respectively [14]. Figures (**a**,**b**) are energy band diagrams for energy level shifting of NBA and MBA treated devices, respectively.

The photovoltaic performance summarized in Table 1 evidenced that the interface modification in nanostructured solar cells with SAMs improves the overall performance of the solar cells [69,70,71] due to the increased tunneling probability which is attributed to reduced effective barrier, and also due to the induced dipole at the donor–acceptor interface which realigns the energy level [72]. Figure 3 explains this behavior by comparing 4-Nitro Benzoic Acid (NBA) and 4-Methoxy Benzoic Acid (MBA). The electron accepting NO_2_ group of NBA leads to a dipole moment pointing towards the TiO_2_ surface. Thus, the work function of TiO_2_ was raised. The electron donor, methoxy group of MBA leads a dipole moment pointing away from the TiO_2_ surface, thus reducing the work function of TiO_2_ [14]. In such a way, SAMs are involved in the shifting of the TiO_2_ conduction band position relative to the highest occupied molecular orbital (HOMO) level of P3HT. This behavior highly influences the interfacial charge separation; thus, the current density (*J_SC_*) was increased. Furthermore, the proper additives of SAMs and the polymer annealing temperature are the crucial factors to enhance the wettability of TiO_2_ and the *V_OC_* of fabricated solar cells.

**Figure 4:**
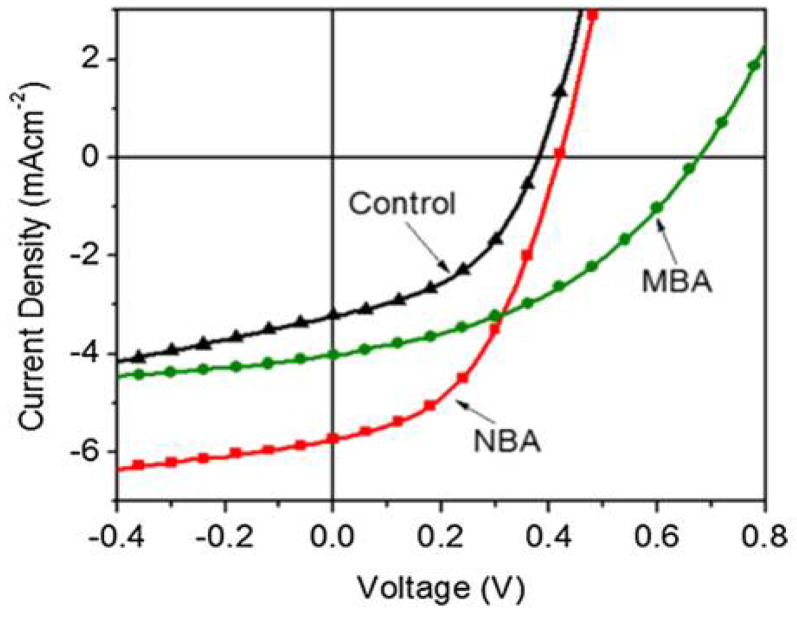
Current density–voltage (J–V) characteristic of the TiO_2_/P3HT solar cell with SAMs under illumination (AM 1.5, 100 mW/cm^2^).

As depicted in Figure 4, the NBA treated TiO_2_/P3HT solar cell showed almost a factor of two increment in the *J_SC_* and slightly improved *V_OC_* whereas the MBA treated cell exhibited significant improvement in *V_OC_*. The insertion of SAMs at the TiO_2_/P3HT interface creates a barrier for back electron transfer (recombination), and, thus, it facilitates a driving force for electron transfer from polymer to TiO_2_. This driving force is evidenced with an increase of the resulting open circuit voltage (*V_OC_*) of fabricated devices. In addition to the energy level alignment at the interface, the molecular orbital of the SAM molecule forms an electronic state at the interface to mediate forward charge transfer or prompt interfacial charge recombination. Further, SAMs can be used to improve adhesion, wettability, compatibility, and charge transfer properties at the interface to reduce interfacial charge recombination [73].

### 2.2. Inorganic Insulating/Semiconducting Layers as Interface Modifiers

Interface modification has also been carried out in hybrid Titanium dioxide/Poly(3-hexylthiophene) heterojunction solar cells using inorganic insulating/semiconducting layers. Table 2 summarizes the work reported in the literature with the inorganic insulating/semiconducting layers and their influence on photovoltaic performance.

The charge transportation at the TiO_2_/P3HT interface is much faster than carrier recombination. In this regard, the deposition of a large band gap owned inorganic insulating thin layer on top of the TiO_2_ surface is a promising modification in order to control carrier recombination by acting as a kinetic barrier at the TiO_2_/P3HT interface [73,74]. The reduced recombination is seen to directly influence the enhancement of *J_SC_* and *V_OC_* of the solar cells.

Loheeswaran et al. reported the photovoltage transients of the TiO_2_/P3HT devices in the presence of Al_2_O_3_ as an interlayer [73]. The lifetimes of the decays were reported as 0.07 ms and 0.5 ms for the control TiO_2_/P3HT and interface modified TiO_2_/Al_2_O_3_/P3HT devices, respectively. This result evidenced that the interfacial recombination at the TiO_2_/P3HT interface is controlled by the interlayering of Al_2_O_3_. Thus, the overall efficiency increased.

Moreover, the improved electronic conductivity and enhanced crystallinity of TiO_2_ morphology and enhanced spectral response of the inorganic insulator material provide further support to enhance the PCE through proper charge collection of the fabricated solar cell. However, most of these inorganic insulators do not participate in carrier generation. This is a primary downside of these materials when they are employed with absorbing polymers like P3HT.

**Figure 5:**
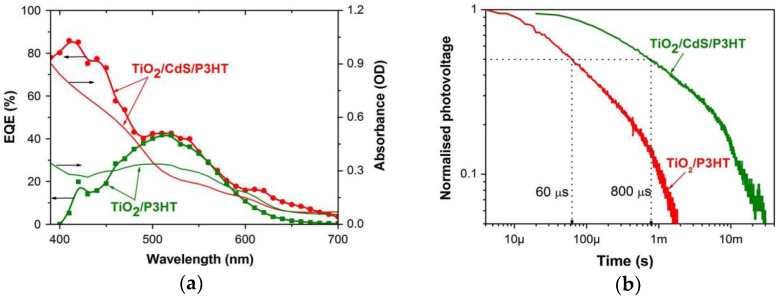
(**a**) External quantum efficiency and absorbance spectra of CdS incorporated TiO_2_/P3HT solar cells and nanostructured films, respectively. (**b**) Double logarithmic normalized photovoltaic transient decay of TiO_2_/P3HT solar cells with and without CdS [79].

The insertion of a semiconducting CdS layer at the TiO_2_/P3HT interface broadens the spectral response as in Figure 5a and controls recombination kinetics as in Figure 5b [79]. Therefore, the overall efficiency was improved by a factor of three. In this study, semiconducting CdS play a dual role, as interface modifier and co-sensitizer in the hybrid TiO_2_/polymer solar cells.

### 2.3. Carbonaceous Materials as Interface Modifiers

There are several carbonaceous materials employed as an interface modifier in hybrid solar cells. Carbon nanotube (CNT) is one of the promising carbonaceous materials that can be used as an interface modifier for hybrid polymer solar cells due to its interesting optoelectronic properties [80]. The recent progress in insertion of carbonaceous materials at the TiO_2_/P3HT interface were analyzed and summarized in Table 3. Carbonaceous materials like multi wall carbon nanotubes (MWNTs) have enhanced number of percolation routes for charge transportation. MWNT incorporating TiO_2_ suppress recombination due to the fast charge transfer through its percolated networks while reducing the electron losses [81]. The proper charge transport at the interface results in enhancement of hole-mobility of the overall nanocomposite and in increase in current density. The surface roughness of MWNT coated TiO_2_ electrodes facilitates the deposition of well-aligned P3HT chains around MWNT due to π-π interaction. However, the increased MWNT wt% could lead to a reduction in the fill factor and *V_OC_*, which is probably due to the shunting by the excess amount of MWNTs.

### 2.4. Small Molecule Sensitizers as Interface Modifiers

**Figure 6:**
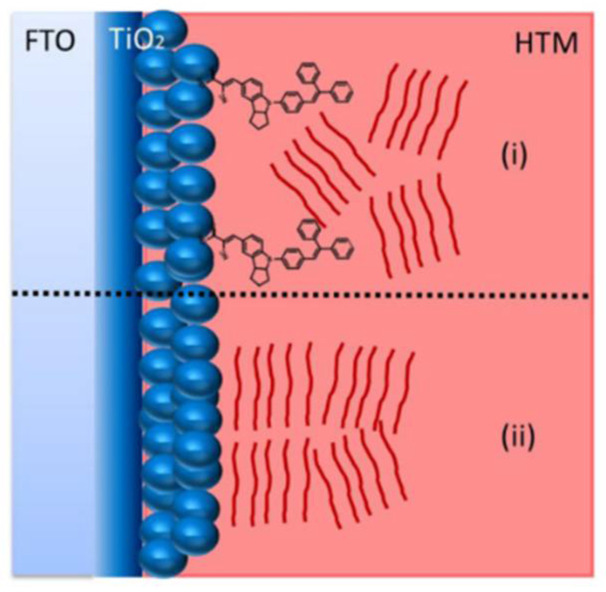
Schematic representation of polymer arrangement in TiO_2_/P3HT HSCs when (**i**) the interface is modified with small molecule sensitizers and (**ii**) unmodified interface.

The insertion of small molecule sensitizers at the TiO_2_/P3HT interface is analyzed and summarized in Table 4. The most common small molecule sensitizers are organic dyes. The cis-Bis(isothiocyanato)(2,2′-bipyridyl-4,4′-dicarboxylato)(4,4′-di-nonyl-2′-bipyridyl) ruthenium(II) (Z907), Di-tetrabutylammoniumcis-bis(isothiocyanato)bis(2,2′-bipyridyl-4,4′-dicarboxylato)ruthenium(II) (N719), Ru(bpy)2(dcbpy)(ClO4)2[(bpy)2,2′-bipy ridine; dcbpy = 4,4′-dicarboxy-2,2′-bipyridine] (RuC), cyanoacrylic acid group ((E)-2-cyano-3-(3′,3″,3‴-trihexyl-[2,2′:5′,2″:5″,2‴-quaterthiophene]-5-yl) acrylicacid) (4T), 2-Cyano-3-[4-[4-(2,2-diphenylethenyl)phenyl]-1,2,3,3a,4,8b-hexahydrocyclopent[b]indol-7-yl]-2-propenoic acid (D131), and 5-[[4-[4-(2,2-Diphenylethenyl)phenyl]-1,2,3-3a,4,8b-hexahydrocyclopent [b]indol-7-yl]methylene]-2-(3-ethyl-4-oxo-2-thioxo-5-thiazolidinylidene)-4-oxo-3thiazolid ineacetic acid (D149) are the notable dyes that played important roles at the TiO_2_/P3HT interface of HSCs. Figure 6 depicts the schematic representation for how the small molecule sensitizers can influence the polymer arrangement in HSCs. They are accomplished as a “bridge” to enrich the exciton dissociation, prevent charge recombination, and enhance the electron lifetime. Dye molecules are the small structures that can easily bind with TiO_2_ structures and their pore filling nature improves the interfacial area between TiO_2_ and P3HT [75]. The pore-filling and proper contact between TiO_2_ and dye molecules can be achieved by the TiO_2_ surface modifications with TiCl_4_, Li-TFSI, H-TFSI, and TBP molecules. Such suitable surface treatments surge the fraction of pore filling, Fermi level shifting of TiO_2_, and the charge conductivity of fabricated HSCs. Further, TiO_2_ induces polymer disorder mainly at a few nanometers away from the TiO_2_/P3HT interface. In this manner, the surface modification takes place with a huge influence on the molecular arrangement in HSCs.

Further, the porosity of spin-coated TiO_2_ prompted an efficient exciton harvesting from the donors/dyes. The higher hydrophobic nature of the interface modifier is much compatible to work with P3HT which leads to result in reduced recombination, improved exciton splitting, and charge separation at the TiO_2_/P3HT interface. The hydrophilic metal-oxide surface modified with a monolayer of dye molecules facilitates a hydrophobic surface on the polymer. Thus, the compatibility was enhanced. This leads to increased polymer infiltration and therefore enhancement of *J_SC_*. Further, the interfacial characteristics can be tailored by balanced dipole effects through the integration of different morphologies, such as TiO_2_-NPs, TiO_2_-QDs, TiO_2_-NRs, TiO_2_-NWs, and TiO_2_-NCs with suitable dyes at the heterojunction interface [48,92].

**Figure 7:**
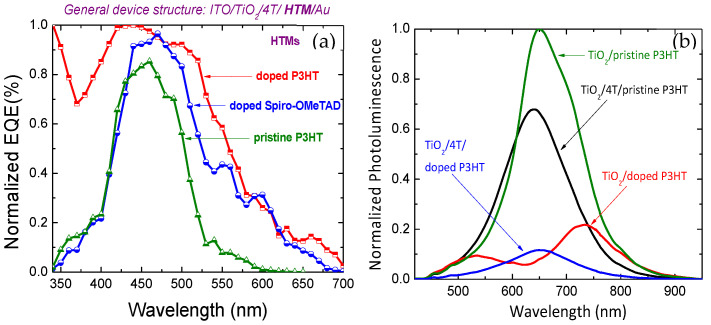
(**a**) Carrier generation and light harvesting properties of ITO/TiO_2_/4T/P3HT or doped P3HT/Au solar cells. (**b**) Photoluminescence quenching of TiO_2_/4T/P3HT and TiO_2_/4T/doped P3HT films attributed due to enriched exciton dissociation via reduced carrier recombination at the TiO_2_/polymer interface.

Apart from these morphological structures of TiO_2_ metal oxides, the molar extinction coefficient, spectral response in the UV region, and functional groups of dye material take the dominant role in the overall carrier generation of HSCs. Higher molar extinction coefficients with wide spectra owned dyes are the most promising sensitizers to work with TiO_2_ and P3HT. In this regard, ruthenium based and thiophene units-based dyes are highly studied in HSCs. Their –COOH anchoring group and tail-like structure of thiophene units can easily penetrate into the mesoporous structures, thus the surface coverage and interfacial interactions will be prompted [30]. The presence of strong electron withdrawing moieties neighboring to the –COOH anchoring group facilitates a molecular dipole directing away from the TiO_2_ surface and enhances the electron affinity of the molecules, and simultaneously *J_SC_* and *V_OC_* [94,96]. Highly enhanced surface coverage and improved interfacial interactions have been reported with the addition of thiophene base molecules rather than –COOH anchoring groups. Moreover, a proper conjugation length of the dye should be used to have enhanced charge generation and collection. Excess conjugation length may discard the interface. The exciton binding energy is reduced by increasing the thiophene chain length. In such cases, the lower conjugation length of dye creates a barrier for charge collection from the P3HT, whereas the highest conjugation length of dyes cannot effectively inject into the TiO_2_ nanostructure. In addition to TiO_2_ nanostructures and dye molecules, the deposition of P3HT is highly concerned. Pre-soaking of dyed TiO_2_ electrodes in P3HT solution prior to the P3HT deposition assists proper infiltration into the nanoporous layer. The combined use of copolymers and additives with P3HT may effectively modulate further enhancement of interfacial interactions between the TiO_2_ and P3HT nanocomposite. On this aspect, recently, both hexyl-substituted thiophene ring based 4T dye and P3HT were employed in titania based HSC along with lithium component as dopant for P3HT, and a high $J_{SC}$ of 13 mA/cm^2^ with highest PCE of 3.95% in dye modified HSCs was reported [95]. This enhanced performance is probably due to the increased charge transport and betterment of light harvesting properties of Lithium doped P3HT along with 4T interface modifier at TiO_2_/doped P3HT interface (Figure 7).

## 3. Summary and Perspectives

In this review, we have focused on the recent progress made and tactics adopted in enhancing the performance of hybrid TiO_2_/P3HT solar cells through interface modifications by employing a range of novel organic and inorganic materials, such as self-assembled monolayers, carbonaceous materials, inorganic insulating layers, and small molecule sensitizers.

It is observed that TiO_2_ induces polymer disorder mainly at the first few nanometers away from the TiO_2_/P3HT interface. Surface modification seems to have a huge influence on the molecular arrangement in HSCs and their performance. A combined study of charge separation, the recombination mechanism, and the polymer disorder will be essential to enhance the PCE of hybrid solar cells. Interlayers can act as a physical spacer between the electron on the inorganic acceptor and the hole on the organic donor. Further, they maximize the electron affinity offset between acceptor and donor in order to promote the exciton dissociation. In summary, the PCE of the HSCs are shown to be highly influenced by the interface modification compared to the crystallinity effect of the TiO_2_ nanostructures. The induced hole transportation and absorption properties of polymer through suitable dopants serve as a backbone for the further enhancement of PCE in interface modified HSC. The combined use of copolymers and additives with P3HT can effectively modulate further enhancement of interfacial interactions between the TiO_2_ and P3HT nanocomposite. The usage of electron rich hexyl-substituted thiophene ring based dyes with P3HT can support better compatibility, high charge transport, and improved light harvesting properties of titania based HSCs. The proper interface modifications with a well-optimized fabrication technique enhance the performance of HSCs and will contribute to studying the principles of the science behind organic photovoltaics.

## Figures and Tables

**Table 1:** Self-assembled monolayers (SAMs) as interfacial modifiers in hybrid Titanium dioxide/Poly(3-hexylthiophene) heterojunction solar cells.

Device Structure	The Function of Interface Modifier on Photovoltaic Performance	η%	Ref.
TiO_2_/NBA/P3HT/PEDOT:PSS	SAMs shift the conduction band position of the porous TiO_2_ relative to the HOMO level of P3HT, and thus influences interfacial charge separation. 4-nitrobenzoic acid (NBA) treatment increases the driving force for electron transfer from polymer to TiO_2_ SAMs act as a barrier or insulating layer for back electron transfer from the TiO_2_ to P3HT.	1.05	[14]
TiO_2_/MBA/P3HT/PEDOT:PSS		1.24	
TiCl_4_ treatment/TiO_2_ nanorod/ACA/P3HT	Reduced back electron recombination Anthracene-9-carboxylic acid (ACA) acts as a linker which provides better compatibility between TiO_2_ and P3HT, and thus, enhances the dissociation efficiency.	0.28	[64]
TiO_2_/4-MP/P3HT	4-mercaptopyridine (4-MP) induces the controlled orientation of P3HT and optimizes the charge separating interface between P3HT and a squaraine dye-decorated TiO_2_. 4-MP+ 4-tert butylpyridine (tBP) enhances the $V_{oc}$ by inducing a conduction band edge shift of the TiO_2_. Thermal annealing of the polymer increases the efficiency by 18.95%	Not reported	[65]
TiO_2_/4-MP+tBP/SQ2/P3HT		1.13	[66]
TiO_2_-quasi-1D/P3HT	Photoelectrode comprises an array of tree-like hyperbranched TiO_2_ quasi-1D nanostructures which were self-assembled from the gas phase. Increased interfacial area of quasi-1D array enhances optical density through increased light scattering, and provides better crystallization of P3HT inside the quasi-1D nanostructure.	1.00	[38]
TiO_2_/P3HT		0.34	
TiO_2_/TAA/P3HT	Improves the wettability of the TiO_2_ surface and P3HT The higher hydrophobic nature of 2-thiopheneacetic acid (TAA) influences the exciton splitting and charge separation	Not reported	[67]
TiO_2_/benzoic acid or 4-nitrobenzoic acid/P3HT	The dipole changes the energy level alignment of the polymer and the TiO_2_. The dipole moment was calculated using DFT as 2.1 D and 3.8 D for benzoic acid and 4-nitrobenzoic acid, respectively.	Not reported	[68]

**Table 2:** Inorganic insulating/semiconducting layers as interfacial modifiers in hybrid Titanium dioxide/Poly(3-hexylthiophene) heterojunction solar cells.

Device Structure	The Function of Interface Modifier on Photovoltaic Performance	η%	Ref.
TiO_2_/Al_2_O_3_/N719/P3HT/PEDOT:PSS	The Al_2_O_3_ coating served as a physical barrier to charge recombination of dye cations. Both $J_{sc}$ and $V_{oc}$ were enhanced and hence the overall efficiency increased by a factor of two.	1.40	[73]
TiO_2_-NR(annealed)/Sb_2_S_3_/ P3HT	The improved electronic conductivity and enhanced crystallinity of TiO_2_ NRs were archived through annealing (500 °C for 2 h) of TiO_2_ NRs prior to the deposition of Sb_2_S_3_.	1.84	[74]
TiO_2_-NR/Sb_2_S_3_/P3HT		1.03	
TiO_2_/CdS/P3HT/PEDOT:PSS	CdS interlayer extended the spectral response, smooth charge transfer, reduced the interfacial charge recombination, and enhanced the built-in voltage	2.40	[75]
TiO_2_/SnS/P3HT	SnS enhanced the charge collection by reducing the loss of electrons, thus, $V_{oc}$ increased.	2.81	[76]
TiO_2_ -NR/CdS/P3HT	Enhanced optical absorption in the visible region resulted an increase in PCE	1.38	[77]
TiO_2_ nanowires/Pyridine/P3HT	Pyridine suppresses back electron transfer recombination at TiO_2_/P3HT interface. CdS contributes to suppress the recombination of the charge carriers.	0.70	[24]
TiO_2_ nanowires/CdS/P3HT		0.45	
TiO_2_/CdS-QD/P3HT	CdS-QDs act as a co-sensitizers	0.87	[78]
TiO_2_/CdS/P3HT	CdS modification enhance the PCE due to increased $J_{sc}$ and $V_{oc}$. CdS layer enhances exciton dissociation and prohibits carrier recombination at the heterojunction, and act as a light absorber at the wavelength around 400 nm.	0.60	[31]

**Table 3:** Carbonaceous materials as an interfacial modifier in hybrid Titanium dioxide/Poly(3-hexylthiophene) heterojunction solar cells.

Device Structure	The Function of Interface Modifier on Photovoltaic Performance	η%	Ref.
TiO_2_-C_60_/P3HT	Incorporation of C_60_ reduced the recombination due to the occurrence of electron transfer from the defect state to the C_60_ percolation network.	0.71	[82]
TiO_2_-Z907/P3HT		0.65	
TiO_2_-C_60_-Z907/P3HT		1.05	
TiO_2_-MWCNT/Z907/P3HT	Increase in hole-mobility resulted when 0.02 wt% MWCNT blended with porous TiO_2_. Enhanced hole-mobility of $\sim 4.510^{-5}{\;\mathrm{cm}}^{2}V^{-1}s^{-1}$ resulted due to the well aligned path ways constructed for charge carriers through incorporation of MWCT with TiO_2_.	Not reported	[83]
TiO_2_-MWCNT/Z907/P3HT	Aligned P3HT chains around MWNT due to p-p interaction. Enhanced efficiency resulted when 0.02 wt% MWCNT blended with porous TiO_2_. The improved performance due to the enhanced number of percolation routes in MWNT, which suppresses back electron transfer via reducing the electron losses. Further increment in MWNT wt% reduces the fill factor and $V_{oc}$, which may be attributed to shunting by the excess amount of MWNTs.	2.50	[81]

**Table 4:** Small molecule sensitizers as interfacial modifiers in hybrid Titanium dioxide/Poly(3-hexylthiophene) heterojunction solar cells.

Device Structure	The Function of Interface Modifier on Photovoltaic Performance	η%	Ref.
TiO_2_/BT5 oligomer/P3HT	Interlayers act as a physical spacer between the electron on the inorganic acceptor and the hole on the organic donor. The electron affinity offset between donor and acceptor should be maximized to enhance the exciton dissociation efficiency.	0.21	[12]
TiO_2_/TiCl_4_/PCBA/P3HT	LiTFSI molecules surges the pore-filling fraction and the charge conductivity for D131-based cells	0.37	[49]
TiO_2_/TiCl_4_/D131/LiTFSI-tBP-P3HT		1.53	
TiO_2_ nanorod/D149/P3HT/PEDOT:PSS	Both carrier generation and recombination at the TiO_2_-NR/D149 and P3HT interface are reduced when TBP molecules are adsorbed on TiO_2_-NR by replacing a few D149 molecules. Fermi level of D149 dye modified TiO_2_-NR is lowered after TBP treatment.	1.58	[84]
TiO_2_ nanorod/D149/TBP/P3HT/PEDOT:PSS		1.83	
TiO_2_-NR/Z907/P3HT/PEDOT:PSS	Z907 and D149 dye molecules provide a proper band alignment and better compatibility between TiO_2_-NR and P3HT, and thus, enhances both charge separation and electron lifetime. Three-dimensional TiO_2_-ND arrays facilitate an increase in the interface area, and thus, a boosted charge separation is observed with D149.	0.94	[85]
TiO_2_-NR/D149/P3HT/PEDOT:PSS		1.98	
TiO_2_-ND/D149/P3HT/PEDOT:PSS		3.12	
TiO_2_/TBP/WL-4/ P3HT	Thiophene end groups of WL-4 improves the mutual compatibility between TiO_2_ and P3HT. Presence of strong electron withdrawing –CN moiety neighboring to the –COOH anchoring group facilitates a molecular dipole directing away from the TiO_2_ surface, and enhances the electron affinity of the molecules, simultaneously enhances the $J_{sc}$ and $V_{oc}$	2.87	[86]
TiO_2_/carboxylated oligothiophene/P3HT	Addition of carboxylated oligothiophenes enhanced surface coverage and improved interfacial interactions.	0.11	[87]
TiO_2_/Z907/P3HT/PEDOT:PSS	The hydrophilic metal-oxide surface modified with a monolayer of Z907 molecules facilitates the hydrophobic surface to the polymer and results in improved compatibility with the polymer. It leads to increased polymer infiltration and therefore enhancement in *J_SC_*. Pre-soaking of the polymer assists proper infiltration into the nanoporous layer.	0.53	[88]
TiO_2_ nanofibers/N719+PPA/P3HT	Improved TiO_2_/P3HT interface resulted with a reduction in the trap state density and suppressed interfacial recombination. PPA is employed with N719 as a co-absorbent	1.09	[89]
TiO_2_ nanofibers/N719/P3HT		0.90	
TiO_2_/N719/P3HT	Interface modification highly influenced the PCE compared to the crystallinity effect of the TiO_2_ nanostructures.	0.35	[22]
TiO_2_/TiCl_4_ treatment/4T/H-TFSI doped P3HT	H-TFSI additive quenches photocurrent generation from excitation of P3HT, but facilitates very effective charge extraction upon excitation of the oligothiophene. The effect of the conjugation length of dye has been studied. The exciton binding energy was reduced by increasing the thiophene chain length. The short chain length owning 1T builds a barrier for charge collection from the P3HT, whereas lengthy chain length owning 5T failed to well inject into TiO_2_. Both oligothiophenes anchored on TiO_2_ and P3HT are involved in photocurrent generation.	1.54	[90]
TiO_2_/TiCl_4_ treatment/5T/H-TFSI doped P3HT		2.32	
TiO_2_/TDCV-TPA/P3HT	Nano porosity of spin-coated TiO_2_ facilitates an efficient exciton harvesting. TDCV-TPA has a facile infiltration into mesoporous TiO_2_ due to its higher absorption coefficients.	0.60	[91]
TiO_2_/TCA/P3HT	Improved exciton splitting and charge separation at the TiO_2_/P3HT interface. The higher hydrophobic nature of TCA is more compatible with P3HT.	0.03	[67]
TiO_2_-NR/P3HT-b-P2VP/P3HT	Copolymer P3HT-b-P2VP effectively modulates the interfacial interactions between the P3HT homopolymer and TiO_2_ nanorod.	1.20	[92]
TiO_2_/LiI+P3HT	LiI induces the photovoltaic response in the ultraviolet region	1.28	[93]
TiO_2_/triphenylamine dye/P3HT	Compatibility enhancement was achieved between TiO_2_ and P3HT. Dye performed as a “bridge” to facilitate the exciton dissociation, inhibit the charge recombination, and enhance the electron lifetime.	2.01	[10]
TiO_2_-NRA/TiO_2_-QDs	Balanced dipole effects tailor the interfacial characteristics through the integration of TiO_2_-QDs and N719 at the heterojunction interface. The tunable device performance resulted with the balanced interfacial dipoles.	0.61	[48]
TiO_2_-NRA/TiO_2_-QDs/N719(4 h)		0.83	
TiO_2_-NRA/TiO_2_-QDs/N719(8 h)		0.91	
TiO_2_/P3HT	The interface modification of dyes improves the hole mobility of the P3HT and involves reduction of recombination at the TiO_2_/P3HT interface. The contribution of thiophene derivative 4T dye in the carrier generation is much higher compared to the standard Ruthenium based dyes. As P3HT has thiophene units, the combination of thiophene derivative dyes with P3HT facilitate a better compatibility than the combination of P3HT with other ruthenium based dyes.	0.41	[30]
TiO_2_/N719/P3HT		0.86	
TiO_2_/Z907/P3HT		1.01	
TiO_2_/4T/P3HT		2.04	
TiO_2_/P3HT	UV–Visible absorption spectra of control TiO_2_/P3HT film is broadened in the UV region in the presence of the RuC dye, and thus carrier generation is high for TiO_2_/RuC/P3HT nanocomposite. Enhanced carrier generation and the extended spectral response evidenced with the extended EQE spectrum. The Photoluminescence quenching and the dark current clampdown of two orders of magnitude reveals that the incorporation of RuC at the interface involves the reduction of recombination.	0.73	[94]
TiO_2_/RuC/P3HT		2.35	
TiO_2_/4T/P3HT	A seminal work with lithium doped P3HT in dye treated hybrid solar cells. Efficiency was significantly increased by doping the P3HT with LiTFSI and tBP. High short circuit current density of 13 mA/cm^2^. Low fill factor values were found due to resulting low shunt resistance. Light harvesting properties of doped P3HT was discussed.	1.04	[95]
TiO_2_/4T/Li doped P3HT		3.95	

## Data Availability

No new data were created in this study. Data sharing is not possible to this article.

## Abbreviations

HSC	hybrid solar cell
4-MP	4-mercaptopyridine
tBP	4-tert-butylpyridine
SQ2	squaraine dye
D131	yellow dye
LiI	lithium iodide
TCA	2-thiophenecarboxylic acid
P3HT	b-P2VP-poly(3-hexylthiophene)-b-poly(2-vinyl pyridine)
PPA	3-phenylpropionic acid
ACA	anthracene-9-carboxylic acid
QDs	quantum dots
AMBIm	2-amino-1-methylbenzimidazole
ND	nanodendrite arrays
MWCNT	Multi Wall Carbon Nanotube
WL-4	cyanoacrylic acid-containing conjugated molecules
TAA	2-thiopheneacetic acid
PCBA	[6,6]-Phenyl C61 butyric acid
NR	nanorod
